# The operations of the free maternal care policy and out of pocket payments during childbirth in rural Northern Ghana

**DOI:** 10.1186/s13561-017-0180-4

**Published:** 2017-11-22

**Authors:** Philip Ayizem Dalinjong, Alex Y. Wang, Caroline S. E. Homer

**Affiliations:** Faculty of Health, University of Technology Sydney, Building 10, Level 7&8, Jones Street, Ultimo, PO Box 222, Sydney, NSW 2007 Australia

**Keywords:** National Health Insurance, Social health insurance, Universal health coverage, Free maternal care policy, Fee exemption, Out of pocket payments, Skilled attendance, Childbirth, Delivery, Ghana

## Abstract

**Background:**

To promote skilled attendance at births and reduce maternal deaths, the government of Ghana introduced the free maternal care policy under the National Health Insurance Scheme (NHIS) in 2008. The objective is to eliminate financial barriers associated with the use of services. But studies elsewhere showed that out of pocket (OOP) payments still exist in the midst of fee exemptions. The aim of this study was to estimate OOP payments and the financial impact on women during childbirth in one rural and poor area of Northern Ghana; the Kassena-Nankana municipality. Costs were taken from the perspective of women.

**Methods:**

Quantitative and qualitative data collection techniques were used in a convergent parallel mixed methods study. The study used structured questionnaire (*n* = 353) and focus group discussions (FGDs =7) to collect data from women who gave birth in health facilities. Quantitative data from the questionnaire were analysed, using descriptive statistics. Qualitative data from the FGDs were recorded, transcribed and analysed to determine common themes.

**Results:**

The overall mean OOP payments during childbirth was GH¢33.50 (US$17), constituting 5.6% of the average monthly household income. Over one-third (36%, *n* = 145) of women incurred OOP payments which exceeded 10% of average monthly household income (potentially catastrophic). Sixty-nine percent (*n* = 245) of the women perceived that the NHIS did not cover all expenses incurred during childbirth; which was confirmed in the FGDs. Both survey and FGDs demonstrated that women made OOP payments for drugs and other supplies. The FGDs showed women bought disinfectants, soaps, rubber pads and clothing for newborns as well. Seventy-five percent (*n* = 264) of the women used savings, but 19% had to sell assets to finance the payments; this was supported in the FGDs.

**Conclusion:**

The NHIS policy has not eliminated financial barriers associated with childbirth which impacts the welfare of some women. Women continued to make OOP payments, largely as a result of a delay in reimbursement by the NHIS. There is need to re-examine the reimbursement system in order to prevent shortage of funding to health facilities and thus encourage skilled attendance for the reduction of maternal deaths as well as the achievement of universal health coverage.

## Background

Skilled attendance at childbirth has been recommended for the reduction of maternal and newborn deaths [1–3]. A 20% reduction in stillbirths or maternal deaths is expected when skilled attendants are utilised during childbirth, given the availability of equipment, drugs and supplies [1]. In Ghana, uptake of skilled attendance at childbirth is a challenge, especially for rural populations and poor families. While urban areas and rich families had a skilled attendance coverage of 87.4% and 90.5% respectively, rural areas and poor families had a coverage of 57.2% and 42.1% respectively [1]. Financial barriers are largely attributable to this phenomenon, as a quarter of Ghanaians are poor, especially rural populations [4].

Making direct out of pocket payments (OOP) can prevent poor households from using maternal health services, including skilled attendance at childbirth [5–7]. Poor households are likely to forgo essential health services when they have to make direct OOP payments. In addition, some households are pushed further into poverty through their use of health services [5]. Empirical evidence points to the positive effects of risk pooling programmes, including fee exemption policies on the use of maternal health services. For example, the implementation of fee exemption policies in particular have led to a decrease in OOP payments for maternal health services [8–12]. Fee exemption policies have also improved the utilisation of skilled attendance at childbirth and reduced maternal deaths [12, 13].

To promote skilled attendance at childbirth and reduce maternal deaths in Ghana, a fee exemption policy to cater for pregnant women in the four poorer regions of Central, Northern, Upper East and Upper West was initially implemented in 2003. In 2005, the exemption was extended to the rest of the country. The prime aim of the policy was to remove direct OOP payments associated with the use of maternal health services. Under the policy, metropolitan, municipal and district assemblies were responsible for the reimbursement of health facilities after the provision of maternal health services to women. Nevertheless, governmental budgetary constraints affected the successful implementation of the initiative [14]. The initiative was reintroduced in July 2008 and referred to as the free maternal care policy, operating under the National Health Insurance Scheme (NHIS). Under the policy, pregnant women are entitled to free of charge registration with the NHIS, free of cost health services and drugs up to the time of childbirth and after (90 days). Following the implementation of the policy, studies have shown an increase in coverage for skilled attendance at childbirth [15–19].

However, studies in Africa and Asia have shown that OOP payments still persisted in the midst of fee exemptions policies, especially for skilled attendance at childbirth [11, 20–25]. This phenomenon is attributed to systemic challenges [14]. In Ghana, the benefit package of the NHIS does not cover the cost of transport and thus pregnant women and expectant mothers would have to pay for transport to and from health facilities under the free maternal care policy. But transport cost is reported to be a major cost driver for the use of maternal health services [21, 26]. Given this background, there is limited knowledge on costs associated with the utilisation of skilled attendance at childbirth under the free maternal care policy in Ghana. This study therefore aimed to estimate OOP payments during childbirth and the financial impact on women under the free maternal care policy of the NHIS in poor rural Northern Ghana. OOP payments were determined from the perspective of women.

## Methods

### Study design

Both quantitative and qualitative data collection techniques were used through a convergent parallel mixed methods design [27]. Convergent parallel mixed methods allows for the convergence or combination of quantitative and qualitative data to ensure a comprehensive analysis of a research question [27]. The study is descriptive in nature. The quantitative component of the study was carried out among women who had given birth in health facilities (facility-based). A structured questionnaire were designed to collect quantitative data including information on OOP payments.

Focus group discussions (FGDs) collected qualitative data among women who had given birth in health facilities. A semi-structured interview guide was developed and used for the FGDs. The quantitative and qualitative data collection took place from March to August, 2016.

### Study area

The Kassena-Nankana municipality is one of three municipalities in the Upper East region of Northern Ghana where the study took place. Over two-thirds of the population are rural, with the majority involved in agricultural activities [28]. The municipality has similar characteristics as other poor areas in other regions of Ghana and Africa. A number of health facilities exist in the municipality including community-based health planning and services (CHPS compounds), clinics, and health centres. The highest point of referral within the municipality is the district hospital located in the capital town, Navrongo. Specifically, the study took place in the district hospital, two (2) health centres and eleven (11) CHPS compounds. These health facilities had at least one midwife and were providing childbirth services in the municipality at the time of the study.

### Study population and sampling

The study population was women who gave birth in health facilities in the study area. The sample size was determined through the formula given by Gorstein et al. [29] for a proportion in a single cross-sectional survey. The processes of sample determination for the study has been shown in [30]. This paper used data from three hundred and fifty-three (353) women who gave birth in health facilities (facility-based).

### Data collection

The survey data was captured through structured questionnaire. The data was electronically collected with the use of SurveyCTO Collect v2.10 software application. Survey CTO Collect allows for data capture, transport and processing on hand-held electronic gadgets like tablets and smart mobile phones. Women were identified and recruited daily as they gave birth and were discharged to go home from the study health facilities. Administering of the questionnaire took between 30 and 45 min. The main investigator supervised the data collection which was carried out by research assistants.

### Questionnaire instrument

The questionnaire collected data on the socio-demographic characteristics of the women. Questions on OOP payments including payments to acquire the antenatal record folder, attend antenatal consultation, have laboratory testing, drugs, and blood transfusion were asked. Women were asked about expenditure on food and transportation to and from health facilities. Expenditure for inpatient health services (admissions) were also solicited. An admission was taken to be a stay in a health facility for a period longer than 12 h. Finally, the study collected data on the source of funding for OOP payments. Indirect costs (that is; opportunity costs due to time lost at health facilities) were not assessed due to the challenges associated with the capturing of such data.

### The qualitative study

The study developed and used semi-structured interview guide for the conduct of the focus group discussions (FGDs) with the women. The guide was initially developed in English, focusing on the utilisation of skilled attendance and OPP payments. The guide was translated by experts into the Kasem and Nankam languages spoken in the study area. Back translations were carried out to ensure accuracy of the translations. Overall, seven (7) focus group discussions (FGDs) were held among women who gave birth in health facilities. To ensure women felt free to speak in the FGDs, health providers were not allowed to be present during the discussions. Membership of the FGDs ranged between 5 and 12.

Permission was obtained from all women for the recording of the discussions and field notes were taken. The FGDs were flexible, allowing the facilitator to follow-up specific areas as well as seek clarifications on emerging issues. Particularly, all participants were encouraged to participate in the discussions. To validate the data, issues discussed were presented back to participants for their confirmation or rejection at the end of the discussion. In addition, new issues emerging from the discussions were added to the guide for the next round of discussions. The discussions ended (signifying data saturation) when participants had no further responses despite prompts and probes from the investigator. Each FGD lasted 45-120 min. All the FGDs were moderated by the main investigator.

### Data analysis and management (quantitative study)

The analysis was carried out in STATA 14, using descriptive statistics to assist understand the background characteristics and other study variables. The OOP payments were classified as direct medical, direct non-medical and hospitalisation (inpatient). The direct medical expenses (outpatient) comprises of the cost of the antenatal folder, consultation charge, laboratory tests, cost of drugs and blood (for cases of blood transfusion). Direct non-medical expenses included the cost of meals and transportation to and from health facilities. The hospitalisation expenses were for costs incurred as a result of hospital admission (inpatient). The expenditure for inpatient health services comprised of a summation of the costs for medical services, laboratory testing, drugs and bedding during admission. The estimation of the overall mean OOP payments was expressed as M = a + b + c, where M = overall mean OOP payments, a = direct medical expenses, b = direct non-medical expenses, and c = hospitalisation cost. The direct medical, direct non-medical and hospitalisation costs were estimated by aggregating the costs from which means and standard deviations were determined.

To estimate the impact of OOP payments on women/households, the overall mean cost was calculated as a percentage of the mean monthly household income of the Upper East region (region of study). Data were not available specifically for the Kassena-Nankana municipality. The average annual household income for the region was GH¢7240.5 (US$3673.8) as reported in the Ghana Living Standards Survey Report Round 6 [28]. This translated to an average monthly household income of GH¢603.4 (US$306.2), which was used for the determination of the impact of OOP payments on income of households. OOP payments that are considered to be catastrophic for families were also estimated using the same average monthly household income (GH¢603.4 = US$306.2). Catastrophic OOP payments are those which disrupt the consumption patterns of households, particularly essential goods and services. Catastrophic OOP payments were considered to have occurred when health expenditure for a given episode were equivalent to or exceeded a set threshold for a household’s resource (income or expenditure). Thresholds often vary, ranging from 5% to 40% [26, 31–33]. This study used a 10% threshold for the determination of catastrophic OOP payments, as done in other studies [33–35]. Women whose OOP payments exceeded 10% of the average monthly household income were classified as having made catastrophic OOP payments.

The costs data for the study was reported in Ghana cedis, but converted into US$, using an exchange rate of US$1 = GH¢1.9708 (2013 exchange rate) as existed in the Ghana Living Standards Survey Report of the sixth round [28]. This is to ensure conformity with the data used from that survey (the Ghana Living Standards Survey).

### Data analysis and management (qualitative study)

All audio tapes were translated verbatim into English. Transcripts and field notes were read a number of times to help understand the patterns in the data before coding. The main investigator also reviewed a number of the audio recordings, comparing to their original transcripts. Any difference observed was corrected before the coding. This activity sought to ensure validity and accuracy in the data collected. A further review of the transcripts was carried out, including hand written notes on the transcripts to bring out important findings. Importantly, a coding structure was adopted based on identified themes and sub-themes in the data which was presented in tabular form. A review of the identified themes were done, in comparison with the data sections from which these themes emerged to further ensure validity and accuracy. Changes were made where necessary. Thus the presentation of the findings reflect the themes and relevant quotes from the women.

## Results

### Socio-demographic characteristics and OOP payments by women

Three hundred and fifty-three (353) women who gave birth in health facilities took part in the quantitative aspect of the study. The mean age of the women was 27 years, with the youngest being 16 and the oldest 45 years. The majority of the women were married (96.3%, *n* = 340), practice Catholicism (49.3%, *n* = 174) and belong to the ethnic group “Kasem” (62.6%, *n* = 221). The age group 20-24 had the highest percentage of women (10.9%, *n* = 10) who reported incurring OOP payments (Table 1). Incurring OOP payments was also common among women who were married (9.1%, *n* = 31), had no formal or basic education (23.7%, *n* = 27), farmers (14.9%, *n* = 18) and first time mothers (8.4%, n = 10). Majority of the women (68%, *n* = 240) gave birth in the district hospital. This was followed by CHPS compounds (19.5%, *n* = 69) and health centres (11.9%, *n* = 42).

**Table 1:** Socio-demographic characteristics and OOP payments by women

Variable		Incurred OOP Payments? *N* = 353		
		Yes	No	Total
		n(%)	n (%)	n(%)
Age	< 20	3 (8.3)	33 (91.7)	36 (10.2)
	20 – 24	10 (10.9)	81 (89.1)	91 (25.8)
	25 -29	8 (6.8)	110 (93.2)	118 (33.4)
	30- 39	7 (7.0)	93 (93.0)	100 (28.3)
	40+	4 (50.0)	4 (50.0)	8 (2.3)
Marital status	Single	1 (7.7)	12 (92.3)	13 (3.7)
	Married	31 (9.1)	309 (90.9)	340 (96.3)
Highest educational level	No formal education	6 (12.8)	41 (87.2)	47 (13.3)
	Basic education	21 (10.9)	172 (89.1)	193 (54.7)
	Secondary/Technical education	2 (3.1)	62 (96.9)	64 (18.1)
	Tertiary	3 (6.1)	46 (93.9)	49 (13.9)
Occupation	Unemployed	1 (4.2)	23 (95.8)	24 (6.8)
	Trader	8 (11.3)	63 (88.7)	71 (20.1)
	Farmer	18 (14.9)	103 (85.1)	121 (34.3)
	Public/Civil servant	2 (4.4)	44 (95.6)	46 (13.0)
	Student	1 (2.6)	37 (97.4)	38 (10.8)
	Other	2 (3.8)	51 (96.2)	53 (15.0)
Religious background	Traditional	4 (6.7)	13 (93.3)	17 (4.8)
	Catholic	9 (6.0)	170 (94.0)	174 (49.3)
	Protestant	16 (12.6)	118 (87.4)	134 (37.9)
	Muslim	2 (7.1)	26 (92.9)	28 (8.0)
Ethnicity	Kasem	15 (6.8)	206 (93.2)	221 (62.6)
	Nankam	15 (14.2)	91 (85.8)	106 (30.0)
	Other	2 (7.7)	24 (92.3)	26 (7.4)
Number of births	1	10 (8.4)	109 (91.6)	119 (33.7)
	2	8 (8.3)	88 (91.7)	96 (27.2)
	3	4 (5.7)	66 (94.3)	70 (19.8)
	4 or more	10 (14.7)	58 (85.3)	68 (19.3)

### Coverage of health expenses by the NHIS during childbirth

In total, 69.4% (*n* = 245) of the women who gave birth in health facilities perceived that the NHIS did not cover all the expenses incurred during childbirth. The women in the FGDs reported that they had made OOP payments for drugs and other supplies during childbirth despite these being covered by the NHIS. A woman said:

*“I paid for my injection during pregnancy and when I delivered the drugs for the baby they wrote it for me to go and buy”* (FGDs, woman).

Apart from payments for drugs and other supplies, women were often given a prescribed list of items by health providers to purchase in preparation for childbirth. These items were to be used during childbirth and after, comprising of disinfectants, soaps, rubber pads, and clothing for newborns as the facilities lacked funds to purchase these items. Most of the women indicated that they bought these items for use during childbirth. A woman reported:

*“They let you buy pad so that when you deliver they will use it, they will also let you buy detol [disinfectant] and all those things are not free. The pad is GH¢10 [US$ 5.10] and the detol is GH¢5 [US$2.50], meanwhile when you are pregnant you don’t work”* (FGDs, woman).

Women underwent financial strain to buy the prescribed items in preparation for childbirth. Women needed to exhaust their savings they would have made before pregnancy or rely on either their husbands or other relatives to purchase the items.

### Estimated OOP payments during childbirth

The study estimated the OOP payments made for childbirth. The majority of women (91.8%, *n* = 324) incurred a mean OOP payment of GH¢ 48.60 (US$24.70) for drugs (Table 2). The mean for direct and direct non-medical expenses was GH¢42.90 (US$21.80) and GH¢34.20 (US$17.40) respectively. Hospitalisation cost averaged GH¢4 (US$2). The total OOP payments during childbirth was a mean of GH¢33.50 (US$17).

**Table 2:** Estimated OOP payments during childbirth

OOP expenditure incurred during childbirth			Mean	Std dev	Min	Max
	No.	% of women	GH¢ (US$)	GH¢ (US$)	GH¢ (US$)	GH¢ (US$)
(a)Direct medical expenses (outpatient)						
Folder fee (antenatal record)	2	0.6	11.50 (5.80)	3.53 (1.80)	9 (4.60)	14 (7.10)
Consultation	–	–	–	–	–	–
Laboratory test	63	17.8	22.73 (11.50)	45.35 (23)	5 (2.50)	300 (152.20)
Drugs	324	91.8	48.64 (24.70)	88 (44.70)	3 (1.50)	876 (444.50)
Blood transfusion	9	2.5	31.27 (15.90)	23.16 (11.80)	5 (2.50)	70 (35.50)
Total direct medical expenses	332	94.1	42.85 (21.80)	74.08 (37.60)	3 (1.50)	876 (444.48)
(b)Direct non-medical expenses						
Food (meals)	264	74.8	22.30 (11.30)	22.22 (11.30)	1 (0.50)	110 (88.51)
Transport	105	29.7	23.44 (11.90)	19.02 (9.60)	2 (1.01)	100 (50.74)
Total direct non-medical expenses	274	77.6	21.82(11.10)	20.17 (10.20)	1 (0.50)	110 (88.51)
Total direct and direct non-medical expenses (a + b)	343	97.2	34.23 (17.40)	57.04 (28.90)	4.5 (2.28)	876 (444.48)
(c)Hospitalisation expenses (inpatient)	33	9.3	3.96 (2)	3.56 (1.80)	2 (1.01)	18 (9.13)
(M) Overall direct expenses (a + b + c)	343	97.2	33.53 (17)	56.98 (28.90)	3.5 (1.77)	876 (444.48)94

Exchange rate of GH¢1.9708 = US$1

### Impact of OOP payments on average monthly household income

The mean for total direct medical expenses was estimated to be 7.1% of the average monthly household income of the region (Table 3). The overall mean for direct expenses associated with childbirth was 5.6% of the average monthly household income of the region. In addition, an estimated 36% (*n* = 145) of the women incurred catastrophic OOP payments, using a 10% threshold of the average monthly household income.

**Table 3:** OOP payments as a percentage of average monthly household income

Average OOP payments	% of monthly household income
Total direct medical expenses	7.1%
Total direct non-medical expenses	3.6%
Hospitalisation expenses	0.7%
Overall direct OOP payments	5.6%

Source of funds for OOP payments during childbirth.

**Fig. 1:**
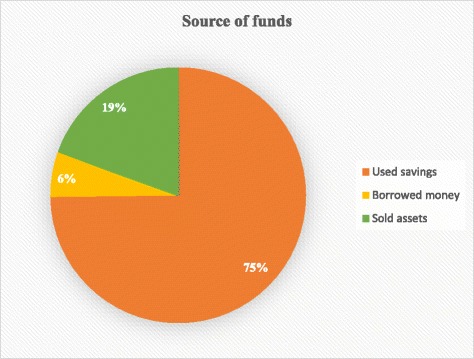
Source of funds for OOP payments during childbirth

Seventy-five percent (*n* = 264) of the women used savings for the payment of the expenses associated with childbirth, whilst 19.0% (*n* = 69) sold assets to finance the payments (Fig. 1).

The findings from the FGDs confirmed that household savings were used to meet the expenditure incurred during childbirth and in some cases, assets in the form of domestic animals and chicken were sold to finance the payments. A woman said:

*“We used all our savings to pay for the cost when I went to the hospital for delivery”* (FGD, woman).

The depletion of savings and sale of assets by women and their families would affect their economic welfare, especially the consumption of essential commodities.

## Discussion

More than two-thirds (69%, *n* = 245) of women who gave birth in health facilities perceived the NHIS did not cover all the expenses incurred during childbirth. They had made OOP payments for drugs and other supplies during childbirth. The women had also bought items consisting of disinfectants, soaps, rubber pads and clothing for newborns. The overall mean for OOP payments during childbirth was GH¢33.50 (US$17) and constituted 5.6% of the average monthly household income for the Upper East region. Approximately, 36% (*n* = 145) of the women incurred catastrophic OOP payments, given a 10% threshold of the average monthly household income. Furthermore, 75 % (*n* = 264) of the women used savings and 19% (*n* = 69) sold assets to finance the payments.

### Buying of drugs and other supplies during childbirth

Over two-thirds (69%) of the women indicated that the NHIS did not cover all expenses and that direct OOP payments were made for drugs and other supplies. Findings from recent studies are consistent with our finding [36, 37]. A qualitative study in Morocco demonstrated that women and their families paid for drugs and other supplies during childbirth in three public hospitals, despite the existence of a fee exemption policy [38]. In Ghana, under an earlier fee exemption policy implemented in 2003, women and their families were found to have made payments for drugs and other services during childbirth [14, 39]. Inadequate government budgetary allocations affected the operations of the previous fee exemption policy [14]. However, the current policy is suffering a similar fate. The OOP payments occurred largely due to stock-outs of drugs and supplies caused by a delay in the reimbursement of health facilities by the NHIS. Consequently, health facilities were unable to procure drugs for dispensation and hence prescription forms were given out to women to purchase drugs out of health facilities. This problem is compounded for the smaller health facilities like the CHPS compounds and health centres with smaller budgets and limited internal generated funding. Another factor triggering the OOP payments is the prescription of drugs outside the essential drug list of the NHIS requiring women and their families to buy. Generally, any OOP payments affected the use of skilled attendance for childbirth [5–7].

### Buying of other items for childbirth

Women and their families were prescribed items comprising of disinfectants, soaps, rubber pads and clothing for newborns to be acquired for use during childbirth. Other studies under fee exemptions policies have had similar findings [24, 40]. Since these prescribed items are not covered by the NHIS and hence women and their families would have to provide them from their own resources, their procurement could affect the utilisation of skilled attendance at childbirth, especially for poor women. Women, especially the poor who cannot afford to buy such items may not be motivated to visit formal health facilities for childbirth and would prefer to give birth at home or elsewhere not requiring the purchase of such items [40]. This might explain why poor women as compared to rich ones were underutilising maternal health services in Ghana [41].

### Mean cost for OOP payments during childbirth

The estimated mean for OOP payments during childbirth was GH¢33.50 (US$17), conforming to previous studies [21, 23, 37, 42–44]. For instance, in rural Tanzania, Kruk et al. reported that 73.3% of women still incurred OOP payments despite a free childbirth policy [21]. The average cost incurred during childbirth in her study was US$5.00 [21]. Another study in North Western Nigeria showed that women and their families spent an average of US$3.00 for normal childbirth [42]**.** The high costs of drugs might help explain the high mean cost for childbirth in our study. A mean cost of GH¢48.6 (US$24.7) was recorded for drugs alone in our study. Compared with most countries in the world, the cost of drugs are very high in Ghana [45]. Given this background, women and their families may be discouraged from using skilled attendance at childbirth, if consistently they have to buy drugs at a very high cost.

Additionally, the overall mean OOP payments recorded during childbirth is considered as high, in the midst of the free maternal care policy. The finding confirmed a study in three African countries which demonstrated that OOP payments for maternal health services constituted a significant percentage of household income, despite some interventional policies being in place in those study areas [46]. A 5.6% expenditure for childbirth alone, could adversely affect the welfare of households in terms of their expenditure on other necessities. This is further supported by the finding that 36% of the women incurred catastrophic OOP payments. The women and their families would have to forgo the consumption of some essentials, for using skilled attendants. This is a de-motivator for the utilisation of skilled attendance for childbirth and the drive for achieving universal health coverage. But universal health coverage is taken as a top priority for the new Director-General of the World Health Organisation, Tedros Adhanom Ghebreyesus, who declared in his first press conference that “All roads should lead to universal health coverage” [47].

### Use of savings and sale of assets for the OOP payments

Despite the existence of the free maternal care policy, our study indicated that 75 % of the women who made OOP payments used savings, with 19% of them selling assets to finance the payments. The finding is consistent with literature [21, 33, 37, 38, 48]. In rural Ethiopia, households resorted to borrowing from relatives and friends and the sale of assets to meet health expenditure for childbirth [48]. Households in rural Bangladesh also used income and savings to make payments for childbirth services [33]. Given the implementation of the policy, women are exempted from paying some of the costs associated with the use of maternal health services, however the recorded payments is seen as burdensome since some coping strategies (savings and sale of assets) had to be adopted. The use of savings as well as sale of assets by women and families for payment of maternal health services could erode the asset base of families. It could also increase the vulnerability of such households to more economic challenges. Thus the systemic challenges requiring the need for OOP payments by the women and families has to be re-examined in order to prevent the perpetuation of the OOP payments. There is the need for health facilities to be adequately resourced, especially under the NHIS. This would minimise or eliminate OOP payments and subsequently promote the use of skilled attendance at childbirth, leading to reduced maternal mortality.

### Study limitations

The study has shown that OOP payments existed during childbirth despite the operations of the free maternal care policy, however, some limitations of the study must be noted. Firstly, since the interviews were carried out after the discharge of women and family members from health facilities to go home, there is the possibility of recall bias, especially on costs. Details of health payments might not be accurately recollected and given out during the interviews. Secondly, the study did not measure the opportunity cost (lost income for time spent seeking health care) associated with the use of skilled attendance during childbirth. These costs were not determined due to the challenges involved in their quantification. The exclusion of opportunity cost affects the overall mean for OOP payments and this should be noted when reading this paper. The study was conducted in only one municipality. There are a total of 216 metropolitan, municipal and district areas in Ghana. As such, the results might not be generalisable to the rest of Ghana, although they would be reflective of many similar regions in Ghana and across Africa.

## Conclusion

The free maternal care policy has not been fully effective in eliminating financial barriers associated with the utilisation of skilled attendance at childbirth. Women and their families continued to make OOP payments for drugs, supplies and other prescribed items during childbirth. This comes in the face of systemic challenges, particularly the delay in reimbursement by the NHIS, leading to stock-outs of drugs and supplies for health facilities. The OOP payments impacted the welfare of the women and households as they used savings and sale of assets to meet the payments. There is the need to re-examine the reimbursement of health facilities by the NHIS in order to prevent shortage of funding to health facilities. When health facilities are well resourced, OOP payments would be reduced or eliminated, leading to increased utilisation of skilled attendance at childbirth and ultimately reducing maternal mortality and the achievement of universal health coverage in the long-term.

## Abbreviations

FGDs: Focus Group Discussions
NHIS: National Health Insurance Scheme
OOP: Out of Pocket
